# Second-Generation Implants for Load Introduction into Thin-Walled CFRP-Reinforced UHPC Beams: Implant Optimisation and Investigations of Production Technologies

**DOI:** 10.3390/ma12233973

**Published:** 2019-11-30

**Authors:** Benjamin Kromoser, Oliver Gericke, Mathias Hammerl, Werner Sobek

**Affiliations:** 1 Institute of Structural Engineering, University of Natural Resources and Life Sciences, Peter-Jordan-Straße 82, 1190 Vienna, Austria; mathias.hammerl@boku.ac.at; 2 ILEK—Institute for Lightweight Structures and Conceptual Design, University of Stuttgart, Pfaffenwaldring 14, 70569 Stuttgart, Germany; oliver.gericke@ilek.uni-stuttgart.de (O.G.); werner.sobek@ilek.uni-stuttgart.de (W.S.)

**Keywords:** UHPC, CFRP, CFRP reinforcement, thin-walled concrete, load introduction in thin-walled concrete elements, metal implants for engineering structures, sustainable construction

## Abstract

Combining two high-performance materials—ultra-high-performance concrete (UHPC) as the matrix and carbon-fibre-reinforced composites (CFRP) as the reinforcement—opens up new possibilities for achieving very lightweight thin-walled concrete elements. This strategy, however, leads to a higher degree of material utilisation, resulting in the generation of higher forces around load introduction points and supports. The authors present a solution for increasing the performance of supports of very slender CFRP-reinforced UHPC beams by using metal implants. Implants are used in place of concrete in regions of stress concentrations and significant deviation forces. These are able to transfer high stresses and forces efficiently due to their ability to sustain both tension and compression in equal measure. A key issue in their development is the interface between the reinforced concrete and metal implant. Building on previous research, this paper deals with the conceptual design of three types of implants manufactured from different metals and with three different types of automated production technologies (water-jet cutting, metal casting with a 3D-printed plastic formwork and binder jetting of steel components). For this paper, tests were carried out to determine the load-bearing behaviour of beams with the three different types of support implants used for load introduction at the supports. A carbon rod served as bending reinforcement and a pre-formed textile reinforcement cage served as shear and constructive reinforcement.

## 1. Introduction

**Figure 1:**
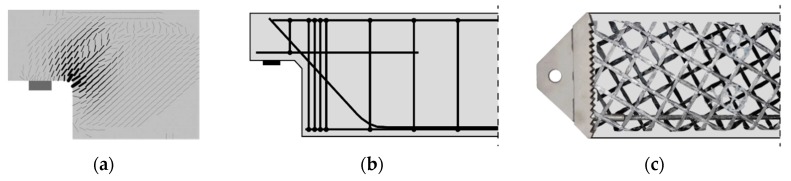
(**a**) Crack distribution of a dapped-end beam subjected to self-weight and uniformly distributed load; (**b**) steel-reinforcement layout of a dapped-end beam [2]; and (**c**) implant for a discontinuity region with carbon textile reinforcement and carbon rod [1].

The material combination of ultra-high-performance concrete (UHPC) and carbon-fibre-reinforced polymers (CFRP) allows the creation of lightweight high-performance building components Kromoser et al. [1]. However, such highly utilised components are sensitive to the turbulent stress fields that were found by and Schlaich et al. [2] to occur in regions of load introduction or geometric discontinuities. An example for a failure induced by the peak stresses occurring in these stress fields is given in Figure 1. To avoid unsatisfactory load-bearing behaviour, so-called implants can be used to substitute the concrete in overstressed areas. Based on an invention developed by Sobek et al. [3], implants are designed to uniformly transfer stresses to the structural elements, thus avoiding premature failure and maximising material utilisation. In terms of weight, CFRP reinforcement outperforms common steel reinforcement in two ways: CFRP exhibits both higher strength and a higher strength-to-weight ratio than steel. Secondly, the concrete cover necessary to protect steel from corrosion due to environmental influences can be reduced to a minimum. Carbon fibres require a concrete cover just thick enough to ensure proper bonding to the concrete. Thus, using carbon-reinforced UHPC yields to more lightweight components than steel-reinforced concrete, as shown in Kromoser et al. [4].

When prefabricated in a controlled environment, the efficiency of CRFC-reinforced UHPC components can be increased even more. The controlled conditions as described in fib Bulletin No. 74 [5] combined with the smaller dimensional tolerances result in an overall increase in structural performance. However, as precast concrete components are designed to incur minimum transport and on-site assembly costs in order to increase their economic benefits as described by Steinle et al. [6], structural considerations may need to be subordinated. As a consequence, structural details such as the dapped end of a beam (see Figure 1a,b) will require extensive additional reinforcement to transfer the high occurring stresses, thus diminishing material efficiency.

**Figure 2:**
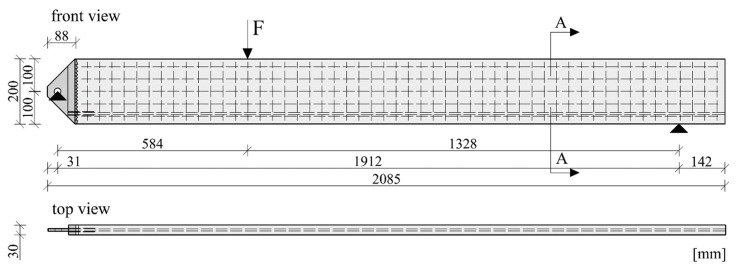
Experimental setup for the tests of the first-generation implants [1].

To avoid excess material usage in areas of stress concentrations in structural elements, researchers of the Institute for Lightweight Structures and Conceptual Design (ILEK) and the Institute for Structural Engineering at the University of Natural Resources and Life Sciences (BOKU) developed metal implants for concrete members [1,3,7,8], which are suitable for various load conditions. The geometry and material of these implants are chosen to ensure the homogeneous transfer of high loads into concrete components, thus avoiding stress concentrations. Furthermore, the high-strength nature of the implant materials allows for point connections, which are easy to assemble and disassemble. Figure 1c shows an implant developed by the authors [1], which was designed to transfer bending and shear forces into a thin-walled concrete beam. In experimental investigations of these implants in combination with a slender CFRP reinforced UHPC beam (test setup shown in Figure 2), the maximum load-bearing capacity of the specimen was determined to be 42.8 kN, thus showing that significant loads can be transferred into the implants with the designed interfaces.

In this paper, the experimental investigations on a second generation of implants for support regions of concrete beams are described. The focus during the further implant development was on efficient structural design, a high degree of automation within the production process, and the use of different metals for the implants. In a first step, a more efficient design (compared to that of the first-generation implants) was developed. Subsequently, three different technologies were chosen to fabricate the second generation of implants: water-jet cutting, metal casting in a 3D-printed formwork and binder jetting of metal components. In addition, various materials were used for the implants: stainless steel, aluminium and stainless steel infiltrated with bronze. One of the main aims was to determine the influence of the material properties on the performance of the various implant types and the interaction between the UHPC and the implants.

## 2. Specimen Design

### 2.1. Previous Research

**Figure 3:**
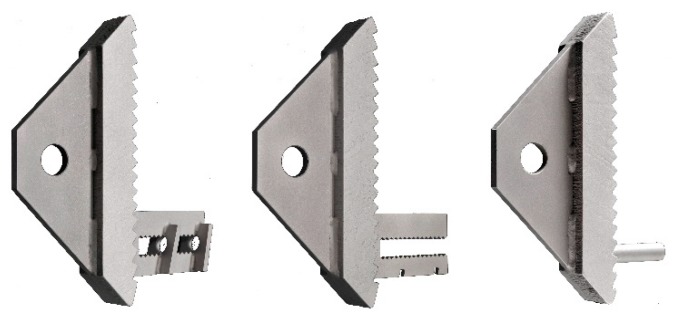
First-generation implants [1].

The research for the first generation of implants for thin-walled CFRP-reinforced UHPC beams aimed at proving the principles of stress-transfer by means of hard contact (compressive stresses) or material bond (tensile stresses). Hard contact was established with a toothed metal bar that ensured load transfer without slippage. One side of each tooth was always oriented perpendicular to acting principal compression forces to avoid large shear forces at the interface. Due to the acting compression forces, the interface would exhibit a frictional resistance (friction coefficient μ=0.57) if shear forces would occur. Tensile stresses in the beam were transferred by a carbon reinforcement bar that was anchored into the implant by means of either a steel-to-concrete bond (Figure 3, left and centre) or by gluing it into a threaded sleeve (Figure 3, right). Except for the M10 threaded sleeve, all components of the implants were laser cut from steel sheets. The imposed geometrical restrictions of this technology resulted in an implant weight of 1550 g. Despite their comparatively high weight, however, all the implants were proven to effectively transfer the loads from the concrete beam to the support. The maximum transferred loads ranged from 25 to 42.8 kN for the test setup shown in Figure 2. A detailed description of the experimental investigations on the first generation of implants can be found in Kromoser et al. [1].

### 2.2. Design Approach

**Figure 4:**
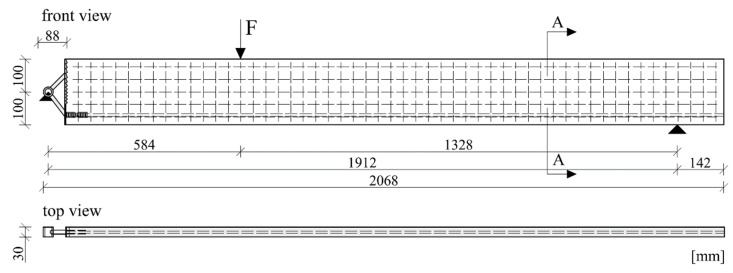
Test setup for the load tests of the second generation of implants.

The first-generation implants were designed to transfer forces from a CFRP-reinforced thin-walled UHPC beam to a bolted support. Both the shear-dominated stress field as the basis for implant design and the experimental setup (Figure 4) of the first-generation implants were also used for the development of the second-generation implants.

As the basic functionality of the chosen principles for load transfer—namely, the hard contact joint for compressive forces and glued connections for tensile reinforcement—had been proven in Kromoser et al. [1], the focus of the presented research was on the creation of more lightweight implants produced with a high degree of automation. Production technologies employed included water-jetting, metal casting with a 3D-printed plastic formwork and binder jetting of the steel components.

The chosen technologies allowed for the production of very filigree components. Therefore, a topology optimisation of the geometry of the first-generation implants was carried out to decrease the required material. To do so, a custom-made Python implementation of the soft kill option method as described by Baumgartner et al. [9] was developed for the FEM software ABAQUS CAE 6.12.

The soft kill option method mimics the adaptive mineralisation of bones by removing material from underloaded regions in structural components in the following steps:

1. An FEM analysis model is generated, and the stress field in the design area is calculated.
2. The Young’s modulus of the material at every mesh element in the design area is adjusted in accordance with the occurring stresses.
3. The stress field of the new component with altered stiffness is calculated.

**Figure 5:**
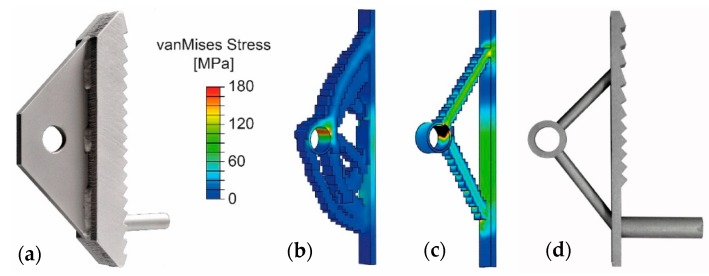
(from left to right) (**a**) First-generation implant with sleeve for reinforcement anchorage; (**b**) intermediate result of topology optimisation; (**c**) final result of topology optimisation (the colour gradient shows the von Mises stresses in MPa); and (**d**) manufactured second-generation implant. The FEA-model used for the optimisation corresponds to the test-setup shown in Figure 2 with a load of F=40 kN.

**Figure 6:**
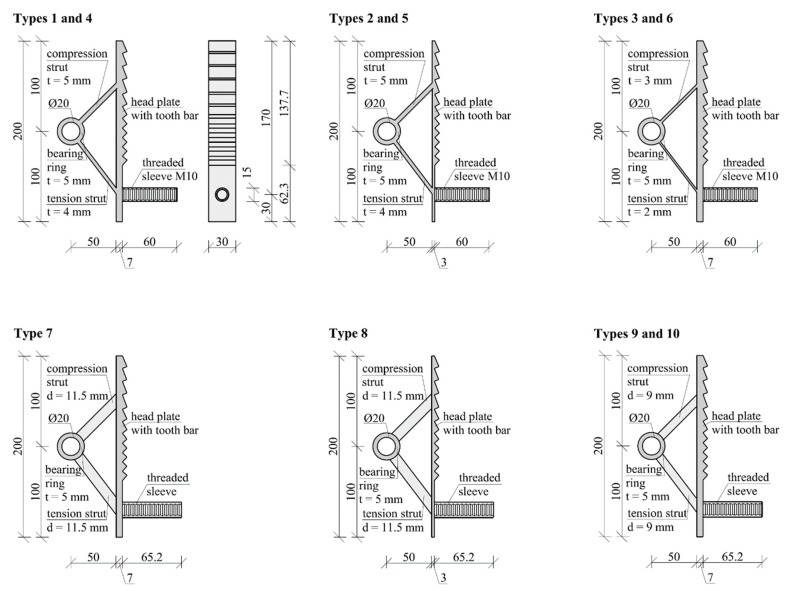
Geometries of all the implant types.

Steps 2 and 3 are repeated iteratively until the Young’s modulus of every element has been reduced to zero or increased to the Young’s modulus of the implant material. Figure 5 shows an intermediate step of the optimisation, as well as the final geometry of the optimised implant. The results of the topology optimisation were subsequently adjusted according to the respective geometrical restrictions of the individual fabrication methods shown in Table 1. Further types of specimens were generated by intentionally weakening individual regions of the implants, specifically the head plate and the struts. This was done to gain additional insight into the failure modes of these parts. The detailed dimensions of the developed parts are given in Figure 6 and Table 2.

### 2.3. Production Methods

**Figure 7:**
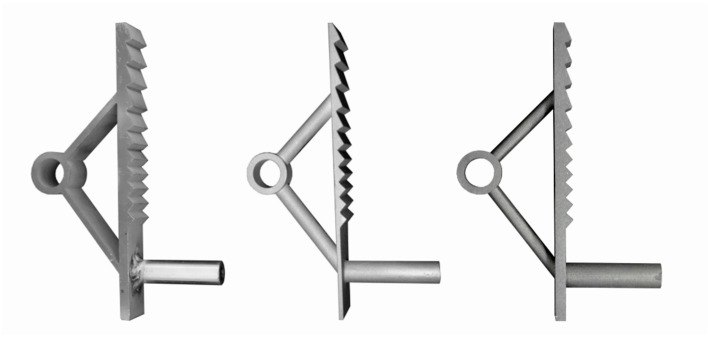
Implant Type 2, water-jet-cut stainless steel (**left**); implant Type 8, steel cast into 3D-printed mould (**centre**); and implant Type 10, binder-jet-printed and subsequently cured steel (**right**).

Three different production methods were used to manufacture the implants. Types 1–6 were produced by water-jet cutting of stainless steel (Types 1–3) and aluminium (Types 4–6). For these implants, sleeves with an internal thread made of the respective material were welded to the tooth bars. The other implant types were fabricated with additive manufacturing methods. Types 7–9 were produced by casting steel in a 3D-printed plastic mould, and Type 10 was produced by directly printing and subsequently curing a steel element of the desired shape. Figure 7 shows examples of implants manufactured with each production method. Each production method has specific characteristics and a different level of automation, which results in different constraints for the design.

#### 2.3.1. Production of Implant Types 1–6—Water-Jet Cutting

Water-jet cutting (also called “abrasive water-jet machining”) is a process in which material is eroded by high-velocity water jets. The advantages include high flexibility, high machining versatility, small machining forces and the absence of both thermal distortion and heat-affected regions. One of the disadvantages is that the striations on the cut surface affect the dimensional accuracy and the quality of the surface finish. A water-jet cutting machine generally consists of a water pumping system, an abrasive feed system, an abrasive water jet nozzle and a catcher. The water and abrasive feed are mixed in the nozzle. After the work piece has been cut with the high-velocity water jet mixed with the abrasive feed, it emerges into a water-filled catcher as described by Aich et al. [10]. The nozzle is moved electromechanically using computerised numerical control. One problem of water-jet cutting is that inaccuracies occur within the processing of thick steel parts. In specimen Types 1–6, the default setting of the jet created notches in the work pieces at the outlet side of the water jet (lower surface of the work pieces). Due to the small thicknesses of the tension and compressions struts, the manufacturing company decided to fill the notches with welds to avoid inaccuracies in the test results. A more professional alternative would be a 3D correction of the water jet and an optimisation of the cutting path. Implant Types 4–6, which were made of aluminium, were able to be cut more accurately due to the easier workability of the material.

#### 2.3.2. Production of Implant Types 7–9—Binder Jetting of Plastic Moulds

Implant Types 7–9 were made of cast steel produced by filling 3D printed plastic moulds. The moulds were produced with a binder-jetting process, in which a binder was printed onto a powder bed layer by layer. The printing nozzle was moved electromechanically using a computerised numerical control. Spherical agglomerates were formed by printing binder droplets onto the powder particles. This ensures an effective bond with the previously printed layers. The printed component remained in the powder bed after printing had completed in order for the binder to set fully and the green component to gain its full strength. The excess powder was subsequently removed by compressed air. This production process could be improved by using several ejection nozzles, which would lead to an increase in speed and a reduction in costs. Any unprinted powder is recycled and can be reused according to Gibson et al. [11]. The components used for implant Types 7–9 were produced by the company Voxeljet (Friedberg, Germany) [12]. Information specific to the produced components can be found in Table 3. The elements produced with the binder-jetting process were used as moulds for investment casting.

#### 2.3.3. Production of Implant Type 10—Binder Jetting of Steel Parts

Implant Type 10 was produced by printing steel in a binder-jetting process. This binder-jetting process is basically the same as that for plastics described above. A binding agent is printed onto a powder bed layer by layer. Each layer is dried by overhead heaters before adding a new layer. The main difference between this process and that used to produce the moulds for implant Types 7–9 lies in the powder material used—steel powder instead of plastic powder is used. After completion of the printing process, the produced component is placed in a curing oven to be sintered together with its surrounding powder. Subsequently, the unbound powder is removed and the so-called green state is infused with bronze, which replaces the binding agent. The produced component now consists of solid metal—approximately 60% steel and 40% bronze (Cu 90% and Sn 10%). Finally, the flutes used to infuse the bronze are removed by hand and the component is polished to obtain a smooth finish. These 3D-printed steel components can also be welded by using TIG (tungsten inert gas) with silicon bronze filler. Table 4 shows product information specific to implant type 10 [13].

### 2.4. Specimen Types

Two main types of implant geometries were developed in accordance with the fabrication constraints identified (see Figure 6). Types 1–6 were designed to be produced by water-jet cutting of metal sheets, which requires a constant cross section over the implant depth. Therefore, a rectangular cross section was chosen for both the tension and compression struts. The only manual step during production was the welding of the threaded sleeve to the implant. Types 7–10 were designed to be produced by direct 3D printing or by casting into a 3D-printed form. A circular cross section was chosen for the compression and tension struts to achieve optimal buckling behaviour. In specimens produced by casting into a 3D-printed form, the blind hole of the threaded sleeve needed to be cut out after production. Specimens produced by direct printing required very little post-processing.

In the tests on the first-generation implants, the failure always occurred in the concrete cross section of the test specimens. To gain a detailed understanding of possible failure modes of the implants in the presented series of experiments, implants with intentionally weakened regions were developed. In some types (Types 3, 6, 9, and 10), the cross-sectional area of the compression and tension struts was reduced. This had a negligible influence on the weight of the implant (see Table 5) and was intended to cause either the buckling of the compression strut or the yielding of the tension strut. Other types were designed with a thinner head plate (Types 2, 5, and 8), which resulted in a weight reduction of the implant of about 30%. The geometry of the tooth bar remained unchanged in all the specimens. Figure 6 gives a more detailed account of the dimensions of the head plates and struts.

## 3. Setup of Experimental Investigations

### 3.1. Test Specimens and Test Setup

The main objective of this test series was to determine the influence of different implant geometries and production methods on the load-bearing behaviour of CFRP-reinforced UHPC beams.

**Figure 8:**
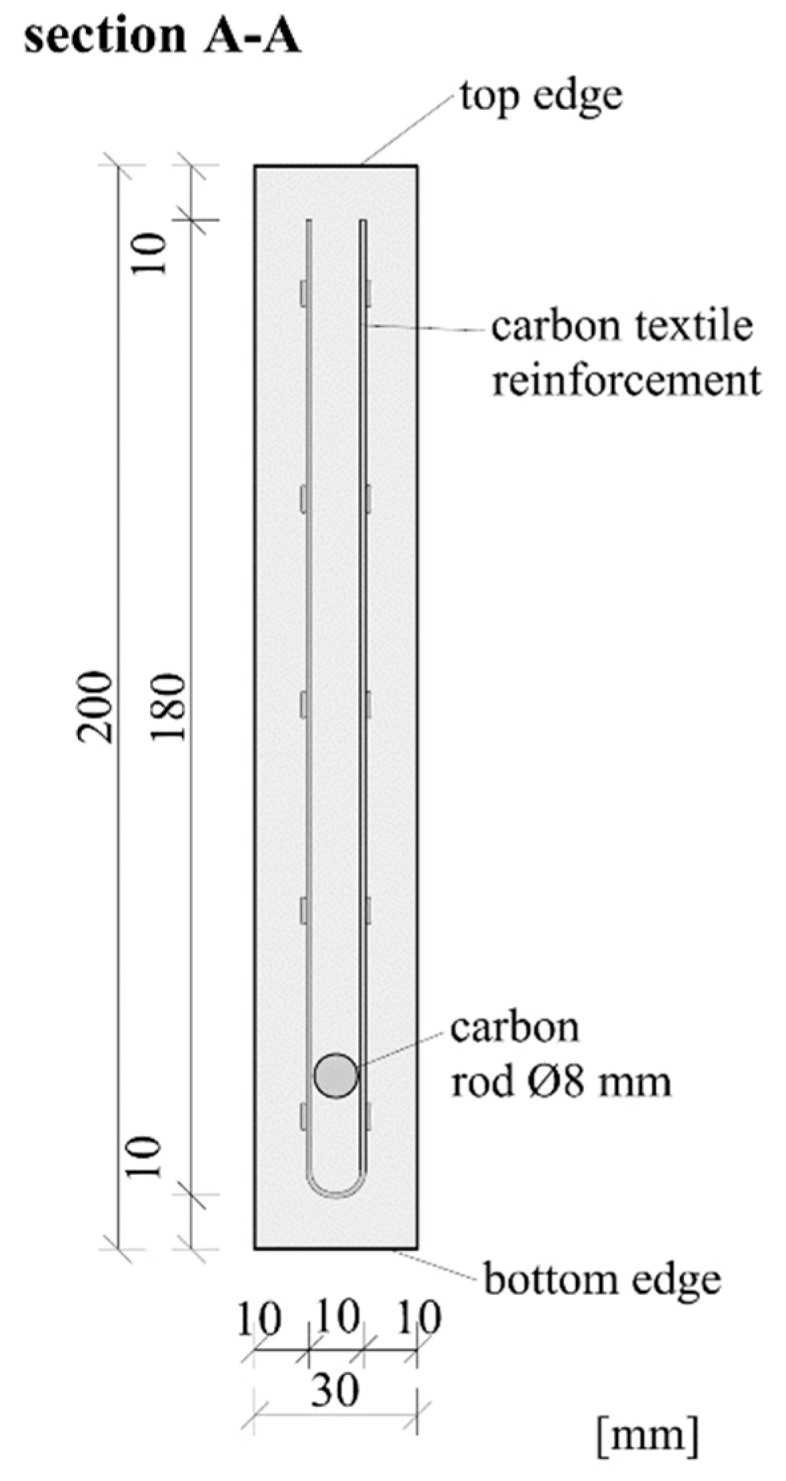
Cross section of the test specimens.

The shear reinforcement of all specimens was U-shaped carbon textile (Q95/95-CCE-38) impregnated with epoxy resin, manufactured by solidian GmbH (Albstadt, Germany). Additionally, a sand-coated carbon rod provided by S&P Handels GmbH (Traiskirchen, Austria) was used as tensile reinforcement. Epoxy adhesive (properties see Table 6) was used to glue the carbon rod inside the threaded sleeve of the implant. A total of ten beams of different configurations were tested. The beams were 2.068 m long, 200 mm high and 30 mm thick. The slender cross section of the test beams and their reinforcement layout can be seen in Figure 8, and the thickness of the specimens measured at the point of load introduction and at the connection with the implant can be seen in Table 7. In all the tests, the load was applied in a displacement-controlled manner, with a load rate of 0.8 mm/min. Linear variable differential transformers (LVDT) were used to measure the deflection of the beams during the tests. One LVDT was situated at the right support of the beam (a fork bearing with an elastomeric pad located between the bottom edge of the beam and the steel bearing), one was fixed at the load introduction point and the remaining two were situated at the left side of the beam so as to measure the deflections at the connection with the implant. A load cell measured the introduced force. The test setup is shown in Figure 4. The area between the implant and the load introduction point was monitored by a non-contact photogrammetric measuring system based on digital image correlation (DIC).

### 3.2. Material Properties

#### 3.2.1. Ultra-High-Performance Concrete (UHPC)

The ingredients of the UHPC mixture are shown in Table 8. Initially, the cement, silica fume, inert additive and quartz sand were mixed for 300 s in a dry-mixing process. Subsequently, water, superplasticiser, slump retainer, a shrinkage-reducing admixture and defoamer were added, and the slurry was mixed for another 150 s. A high-performance mixer was used to provide sufficient mixing energy to uniformly spread and activate the powdery microsilica added to the mixture to improve the properties of the UHPC. The properties of the UHPC mixture are shown in Table 9. Table 10 shows the mean values of the properties of the hardened UHPC. The specimens were 40 × 40 × 160 mm^3^ prisms, as stipulated by EN 196-1, and 100 mm^3^ cubes (informed by the 150 mm^3^ cubes stipulated by EN 206-1). The flexural strength and the Young’s modulus were determined by testing three 40 × 40 × 160 mm^3^ prisms. The material test specimens were treated as the beams (placed beside them in the laboratory) without any cure (dry storage at 20°).

#### 3.2.2. Carbon-Fibre-Reinforced Polymer (CFRP) Reinforcement

Reinforcement made of CFRP has a high tensile strength and is completely resistant to corrosion. The concrete cover can hence be reduced to a minimum and the reinforcement elements are highly durable. CFRP reinforcement is characterised by linear-elastic stress–strain behaviour and brittle failure in tension, and it is sensitive to transverse compression. Currently, there are three different types of CFRP reinforcement available: rods see Sobek et al. [3] and Schumann et al. [14], strands [15] and textile reinforcement [16,17].

CFRP rods and strands are mostly produced by pultrusion. The bundled fibres are pulled through wet resin and brought into the desired shape by extrusion through a die as also described in Schumann et al. [14]. The last step of the production process is the hardening of the structures by heat treatment. The properties of the finished product depend on the properties of the fibres and resin. An important property affecting the usability of this type of concrete reinforcement is the bond behaviour at the interface between the concrete and CFRP. Several surface treatments are available for improving the bond behaviour, such as sand coating the rod, twisting a filament around it or milling a twisted groove into it see Kromoser et al. [4] and Sayed Ahmad et al. [18]. The CFRP rods used for the presented experiments were sand coated. Their properties are shown in Table 11.

Textile reinforcement elements are two- or three-dimensional grids consisting of bundled filaments called rovings [19]. The most common production technology for planar textiles (mass products) is warp knitting, for reasons of production time and economy. Most of the textiles are impregnated with different types of resin to improve the bonding behaviour between the single fibres. The textile reinforcement elements used in the present study consisted of carbon fibres impregnated with epoxy resin. To evaluate the material properties of the textile reinforcement, several tension tests of single strands in the weft and warp directions were performed by the authors. The results are summarised in Table 12.

## 4. Results

In this section, the load-bearing behaviour of the specimens is discussed by comparing their load–deflection curves, failure modes and cracking behaviour. The maximum applied loads and maximum deflections of each specimen are listed in Table 13. The maximum tensile loads at the anchorage were calculated and are also listed in Table 13 to be able to evaluate the connection between the carbon rod and the steel implant for all the test specimens.

**Figure 9:**
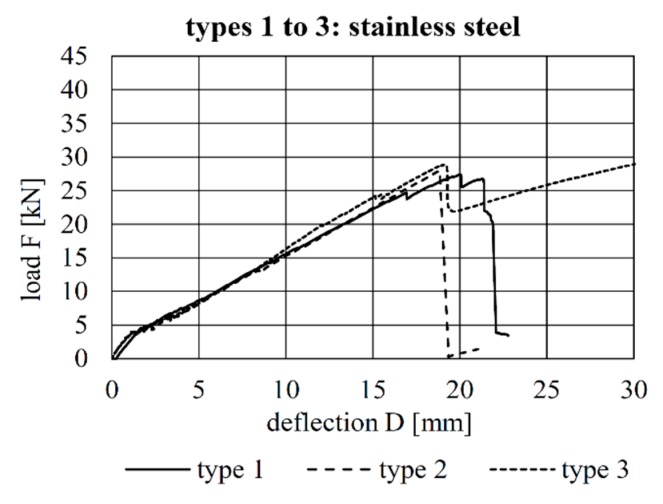
Load–deflection behaviour of the specimens with implant Types 1–3.

**Figure 10:**
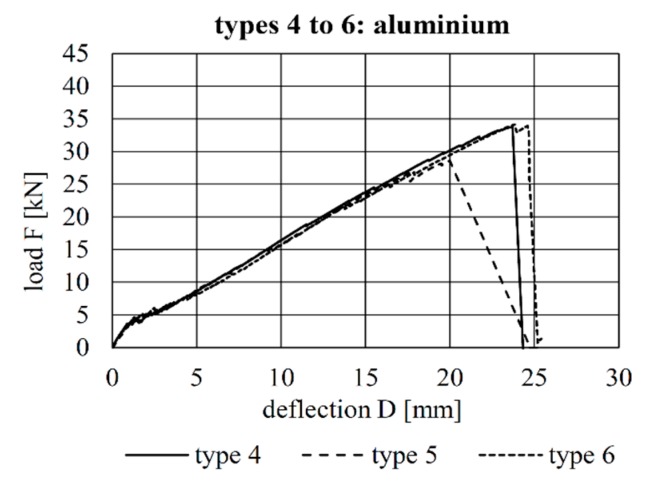
Load–deflection behaviour of the specimens with implant Types 4–6.

**Figure 11:**
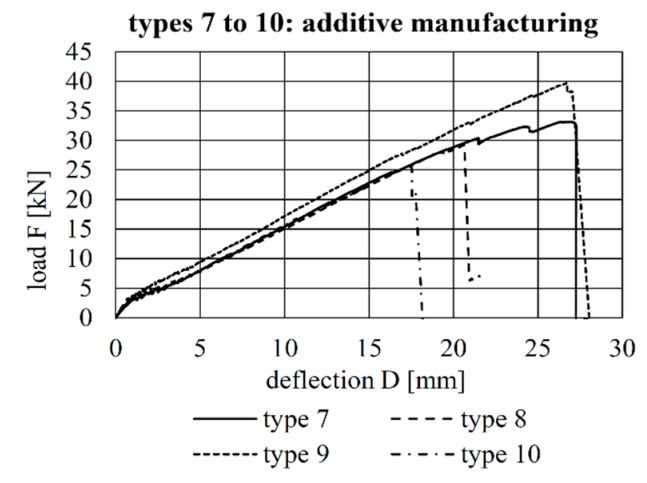
Load–deflection behaviour of the specimens with implant Types 7–10.

**Figure 12:**
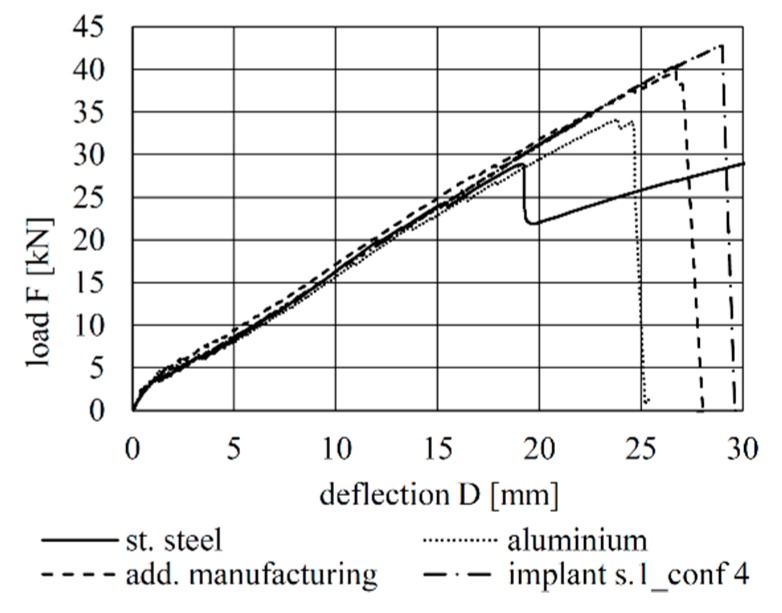
Load–deflection behaviour of the best-performing specimens of the second-generation implants and of the best-performing specimen of the first-generation implants [1].

The load–deflection curves of all tests are shown Figure 9, Figure 10 and Figure 11. A comparison of the load–deflection curves of the best-performing (in terms of structural capacity) second-generation specimens of each production method and that of the best-performing specimen of Configuration 4 of the first implant generation as explained in Kromoser et al. [1] are shown in Figure 12. All curves indicate that three types of structural behaviour occurred during the tests: uncracked (linear-elastic) behaviour, crack formation and stabilised crack formation.

Using the tests of the first-generation implants as reference, it can clearly be seen that the qualitative load–deflection behaviour principally remains unchanged in the second-generation implants. A notable difference between the two generations can be seen during analysis of their failure modes. While failure in the first generation of implants (Type 3—CFRP rods glued into sleeves —see Figure 3) occurred exclusively in the concrete cross section, failure in the second generation also took place in the implants. This is described in more detail in Section 4.3.

In Table 14, the maximum loads sustained by the individual specimens and the weights of the respective implants are compared to the first-generation implant that sustained the highest loads. In comparison to the first generation of implants, the weight of the second-generation implants could be reduced between 55.9% and 89.1%. The load capacities range between 62.2% and 96.8%. This shows a clear increase of the efficiency. The following two implant types exhibited noteworthy structural performance:

- Type 6 (water-jetted aluminium implant with decreased strut cross-section areas) achieved 83.4% of the bearing capacity of the reference, at only 13.8% of the weight.
- Type 9 (investment-cast stainless steel implant with decreased strut cross-section areas) achieved the highest load-bearing capacity (96.8% of that of the reference), at only 40.4% of the weight.

### 4.1. Load–Deflection Behaviour

**Figure 13:**
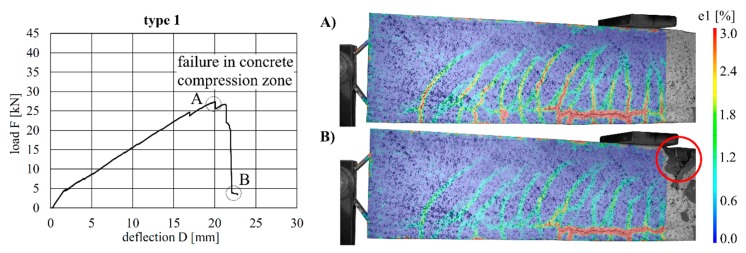
Load–deflection curve of implant type 1 (**left**) and strains (at the beam surface) before (**A**) and after (**B**) failure.

**Figure 14:**
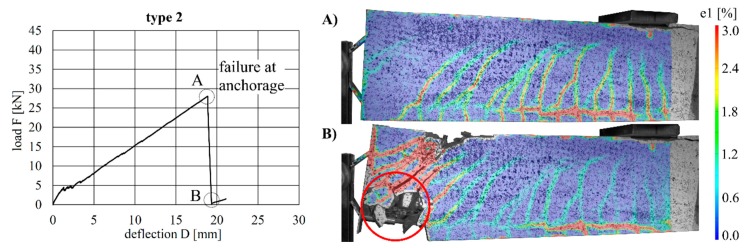
Load–deflection curve of implant type 2 (**left**) and strains before (**A**) and after (**B**) failure.

**Figure 15:**
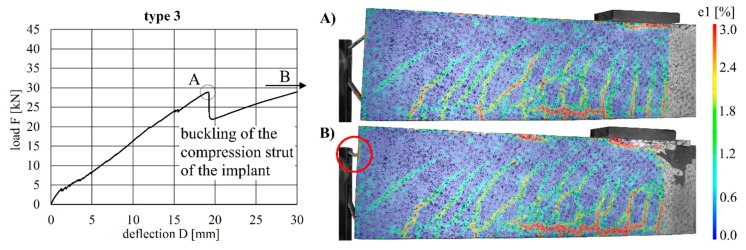
Load–deflection curve of implant type 3 (**left**) and strains before (**A**) and after (**B**) failure.

The load–deflection curves of the beams with implant types 1–3 (stainless steel) and the respective strains at the beam surface are shown in Figure 13, Figure 14 and Figure 15. The maximum deflections were 20.0 mm (type 1), 18.8 mm (type 2) and 35.1 mm (type 3), and the respective maximum loads were 27.4 kN (type 1), 28.0 kN (type 2) and 32.1 kN (type 3).

By considering the load–deflection curves and the strains at the beam surface before and after failure, it can be seen that each specimen exhibits a similar crack formation behaviour. The failure modes were different for each type. type 1 failed in the concrete compression zone (Figure 13), Type 2 failed at the anchorage (Figure 14) and type 3 failed due to the buckling of the compression strut of the implant (Figure 15). It also can be seen that the load of type 3 increased after the implant had failed (at 28.9 kN) and reached a value of 32.1 kN, at which point the beam finally failed due to crushing of the concrete compression zone.

The beams with implant gypes 4–6 (aluminium) all exhibited similar load–deflection behaviour. The maximum deflections were 23.7 mm (type 4), 20.0 mm (type 5) and 23.8 mm (type 6). The maximum loads were 33.8 kN (type 4), 28.6 kN (type 5) and 34.2 kN (type 6).

**Figure 16:**
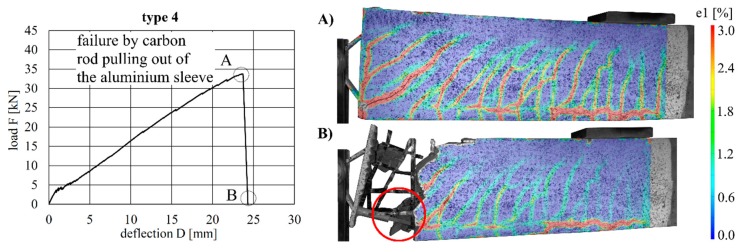
Load–deflection curve of implant type 4 (**left**) and strains before (**A**) and after (**B**) failure.

**Figure 17:**
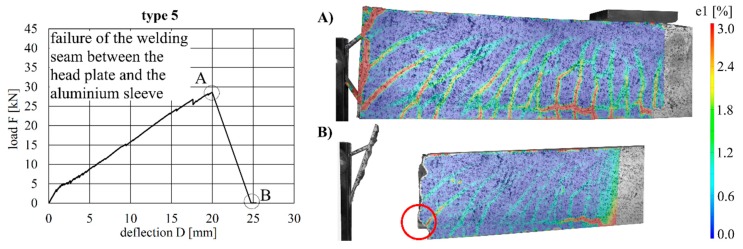
Load–deflection curve of implant type 5 (**left**) and strains before (**A**) and after (**B**) failure.

**Figure 18:**
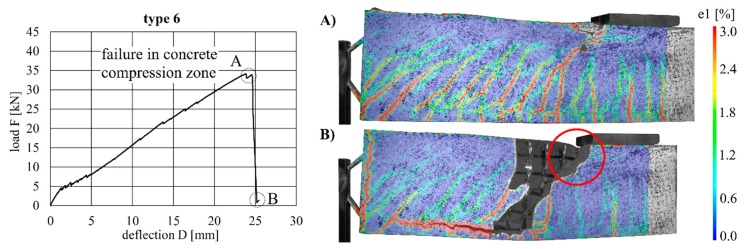
Load–deflection curve of implant type 6 (**left**) and strains before (**A**) and after (**B**) failure.

The load–deflection curves and the crack formation behaviour of the specimens with implant types 4–6 are very similar to each other. Compared to implant type 1 (stainless steel), more shear cracks occurred in the region around the implant, and a crack between concrete and implant was visible in each specimen. One reason for this could be the lower Young’s modulus of aluminium (compared to steel), which leads to a deformation of the implant (Figure 16, Figure 17 and Figure 18). Implant type 4 failed by the carbon rod pulling out of the aluminium sleeve (Figure 16). Type 5 failed at the welding seam between the head plate and the aluminium sleeve subsequent to debonding between the concrete and head plate (Figure 17), which led to a vertical displacement of the concrete beam, as can be seen in Figure 17A. This deformation caused additional vertical forces onto the welded sleeve. This and the resulting moment in the welding seam led to the failure between head plate and aluminium sleeve. Type 6 failed in the concrete compression zone (Figure 18).

**Figure 19:**
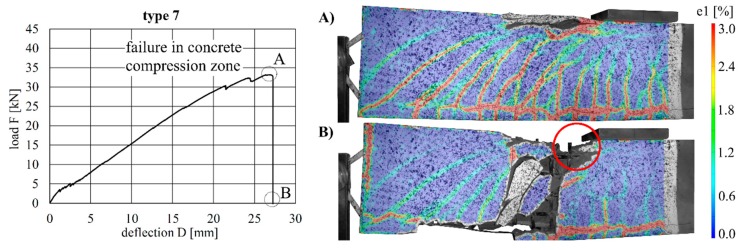
Load–deflection curve of implant type 7 (**left**) and strains before (**A**) and after (**B**) failure.

**Figure 20:**
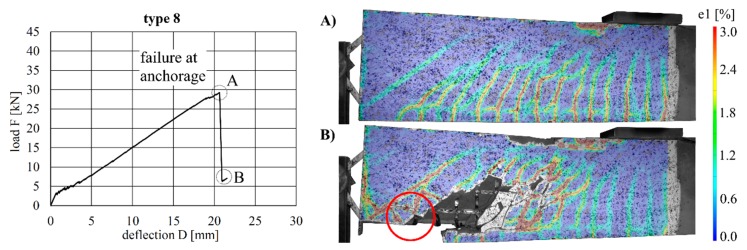
Load–deflection curve of implant type 8 (**left**) and strains before (**A**) and after (**B**) failure.

**Figure 21:**
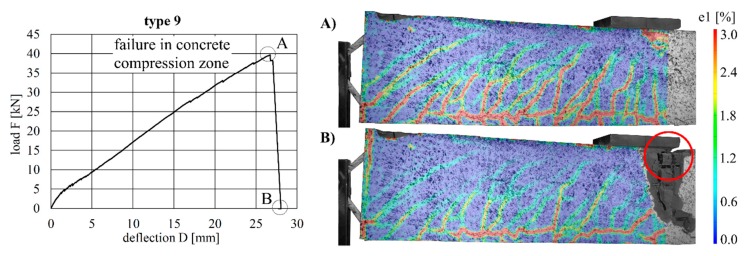
Load–deflection curve of implant type 9 (**left**) and strains before (**A**) and after (**B**) failure.

**Figure 22:**
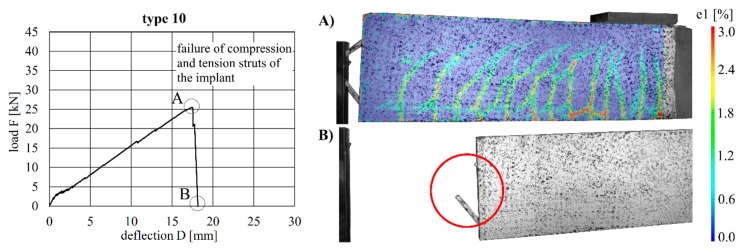
Load–deflection curve of implant type 10 (**left**) and strains before (**A**) and after (**B**) failure.

The load–deflection curves of the beams with implants types 7–10 (produced by additive manufacturing techniques) along with the respective strains at the beam surface are shown in Figure 19, Figure 20, Figure 21 and Figure 22. The maximum deflections were 26.4 mm (type 7), 20.6 mm (type 8), 26.7 mm (type 9) and 17.5 mm (type 10), and the respective maximum loads were 33.2 kN (type 7), 29.3 kN (type 8), 39.7 kN (type 9) and 25.5 kN (type 10).

The load–deflection curve of specimen with implant type 9 indicates that this specimen had a higher stiffness than types 7, 8 and 10. The measurements of the geometry of all specimens (Table 7) show a higher thickness and height at the point of load introduction compared to the other beams. In the specimens with implants made of investment-cast steel, shear cracks similar to those of implant type 2 occurred, but no gap between the implant and concrete could be observed (Figure 19, Figure 20, Figure 21 and Figure 22). types 7 and 9 failed in the concrete compression zone (Figure 19 and Figure 21) and type 8 failed due to the carbon rod pulling out of the sleeve (Figure 20). Type 10 failed due to the rupture of the mounting ring of the implant followed by the failure of the compressive and tensile struts of the implant (Figure 22).

### 4.2. Cracking Behaviour

**Figure 23:**
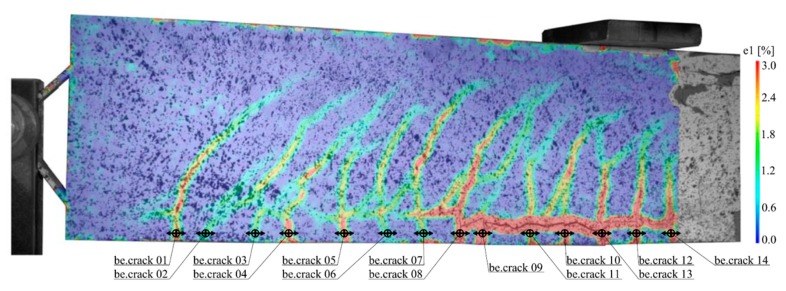
Measurement of the strains and locations of the cracks in type 1 using digital image correlation.

The cracking behaviour of all beams was measured by using digital image correlation. Figure 23 shows for example an evaluation of the test specimen with implant type 1. The bending cracks (be.crack XX) were measured 1 cm above the bottom edge of the beam. The number of bending cracks, the mean crack widths and the mean distances between the cracks just before failure were determined for all the specimens.

**Figure 24:**
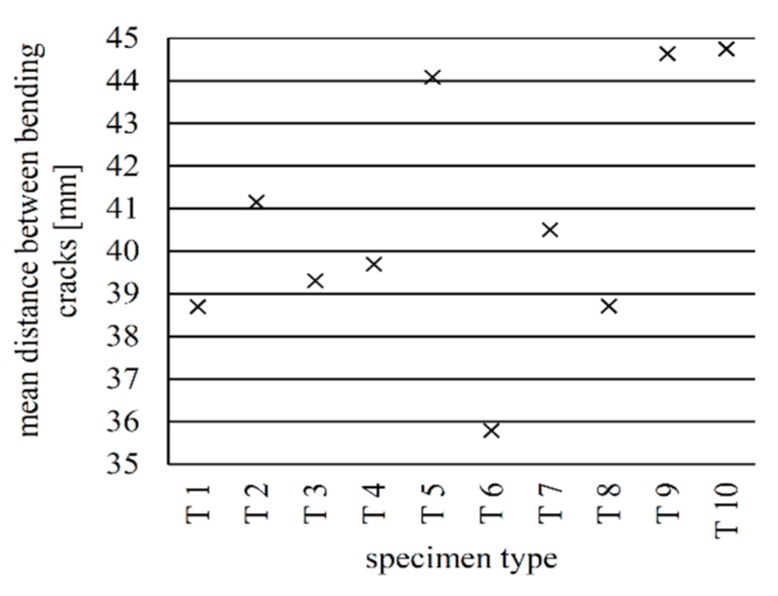
Mean distances between bending cracks at maximum load for all implant types.

Figure 24 shows the mean bending crack distances of all specimens just before the failure occurred. They range between 35.8 mm (type 6) and 44.8 mm (type 10).

**Figure 25:**
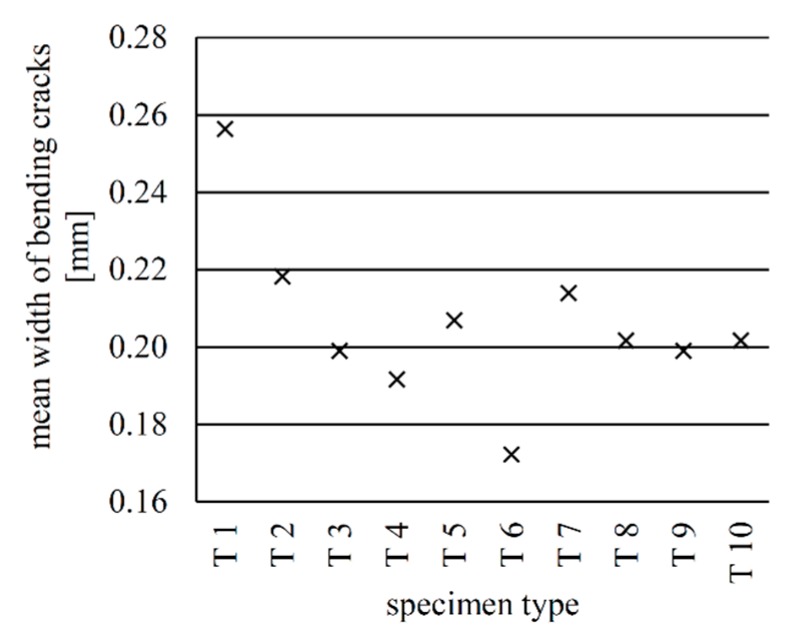
Mean widths of the bending cracks at a load of 24 kN.

The mean widths of the bending cracks of all specimens at a load of 24 kN is shown in Figure 25. Except for types 1 and 6, all specimens exhibited similar crack widths (between 0.19 mm and 0.22 mm). The strains at the surface of type 1 just before failure (Figure 13) indicate that the bond cracks influence the number and consequently the width of the bending cracks. Figure 18 shows that type 6 had a very consistent crack distribution, which explains the low mean width of its bending cracks compared to the other beams.

### 4.3. Failure Modes

The following failure modes occurred during the experiments: failure of the reinforced concrete beam (types 1, 6, 7, and 9), failure at the anchorage (types 2, 4, 5, and 8) or failure of the compression or tension struts of the implants (types 3 and 10).

**Figure 26:**
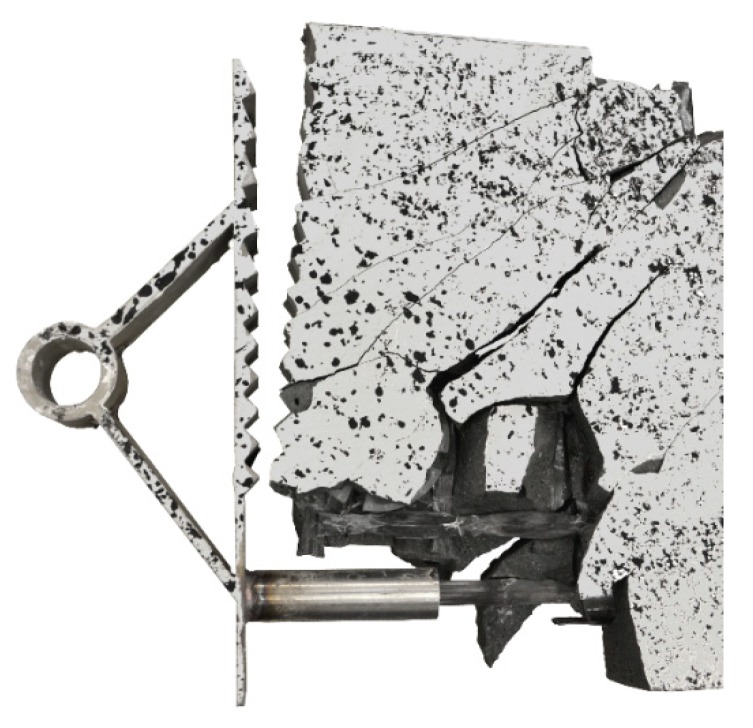
Failure mode of implant type 2—pulling out of the carbon rod from the anchorage.

**Figure 27:**
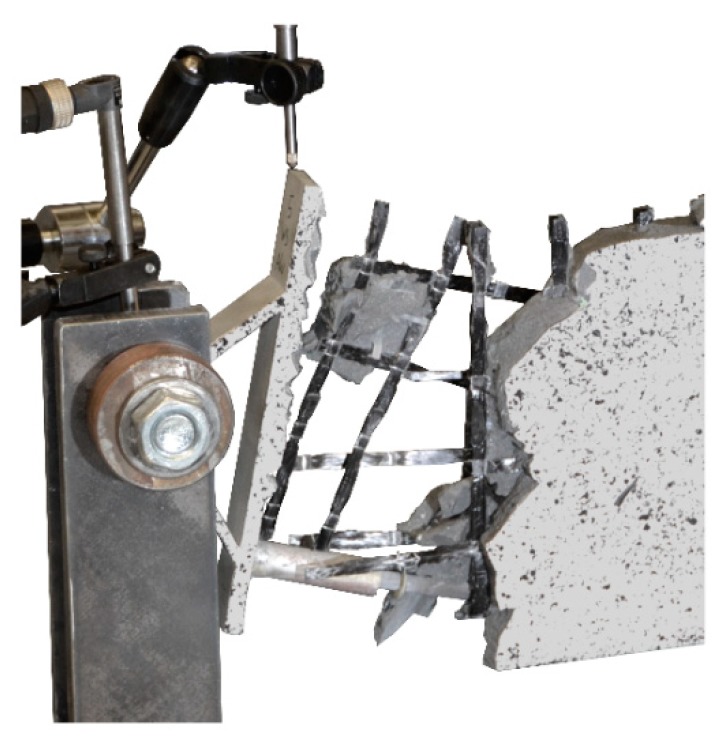
Failure mode of implant type 4—pulling out of the carbon rod from the anchorage.

The failure of types 2, 4, and 8 occurred as a result of pulling the CFRP rod out of the steel sleeve, which serves as end anchorage, at loads of 28.0 kN (type 2), 33.8 kN (type 4) and 29.3 kN (type 8). Figure 26 and Figure 27 show the specimens with implant types 2 and 4 after failure. There, the failure occurred at tensile forces in the CFRP bar at the anchorage of 8.6 kN (type 2), 10.4 kN (type 4) and 9.0 kN (type 8). This shows the importance of the high requirements for the manufacturing of the connection between the carbon rod and the steel implant.

**Figure 28:**
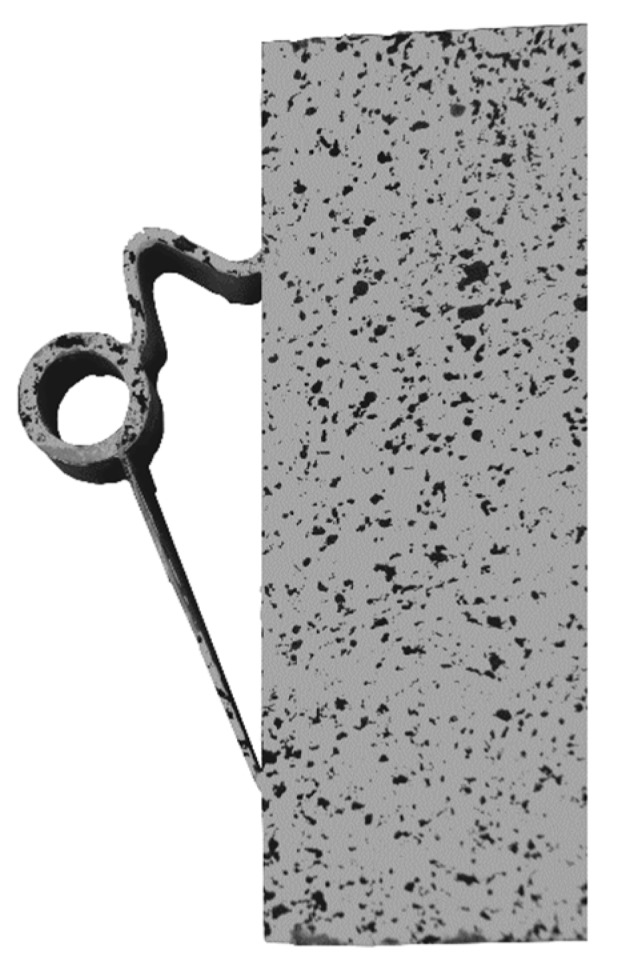
Failure mode of implant type 3—buckling of the compression strut and yielding of the tensile strut.

The failure of implant type 3 occurred due to buckling of the compression strut and subsequent yielding of the tensile strut. This was the only specimen that exhibited a ductile failure mode. After the onset of failure at a load of 28.9 kN, the beam still exhibited residual load capacity. The test was stopped eventually due to the large deformations observed at the maximum load of 32.1 kN. Figure 28 shows the test specimen with the deformed implant after unloading.

**Figure 29:**
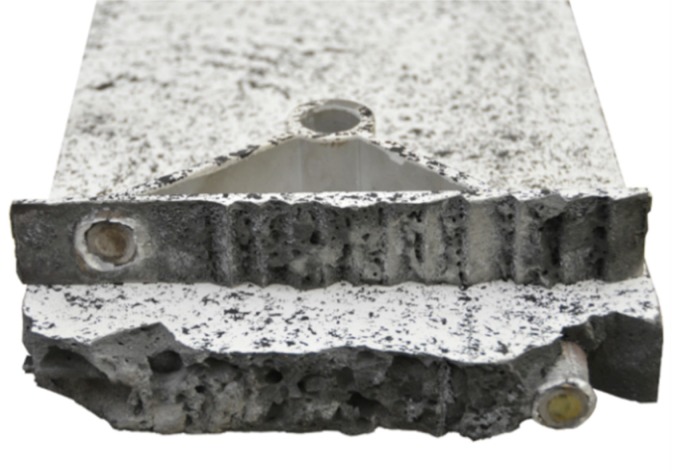
Failure mode of implant type 5—failure of the weld seam.

**Figure 30:**
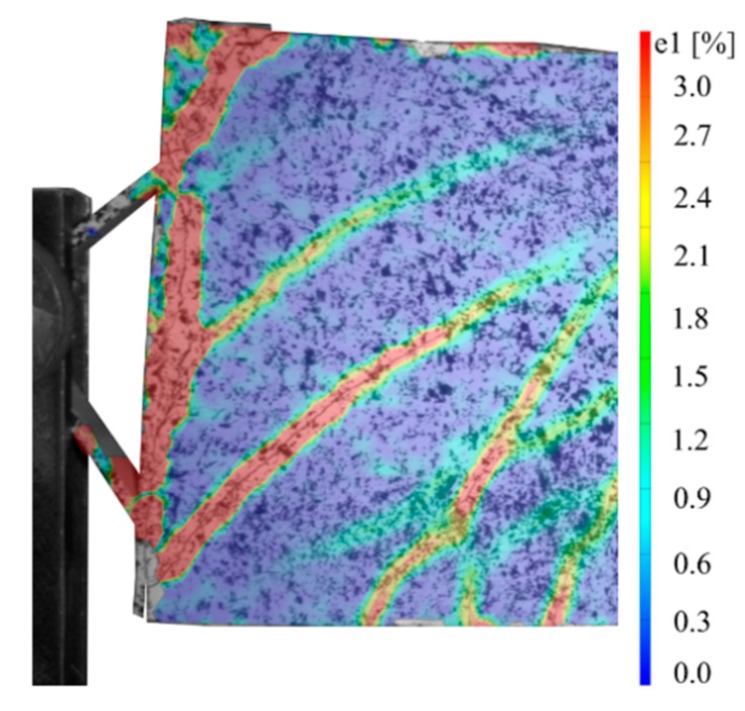
Implant type 5—strain field at the surface of the beam just before failure occurred at a load of 28.6 kN.

Implant type 5 failed due to overstressing of the weld seam between the sleeve and the implant (see Figure 29). The implant was characterised by a thin tooth bar. At a load of 26.7 kN, a crack opened at the interface between the implant and the UHPC beam. Subsequently, the CFRP rod started to pull out of the steel sleeve, which led to the formation of a direct compression strut of the concrete on the sleeve. This induced additional stresses in the welding seam, which led to overstress. Failure occurred at a load of 28.6 kN. Figure 30 shows the strains at the beam surface at the intersection between the implant and the UHPC just before the failure of the specimen.

**Figure 31:**
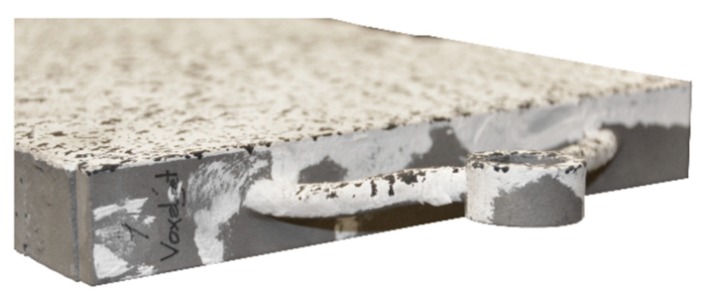
Failure mode of implant type 7—deformed compression strut of the implant.

The test specimen with implant type 7 failed in the concrete compression zone at a maximum load of 33.2 kN. Failure was induced by the buckling of the compression strut of the implant, which commenced at a load of 30.4 kN. The deformed implant is shown in Figure 31.

**Figure 32:**
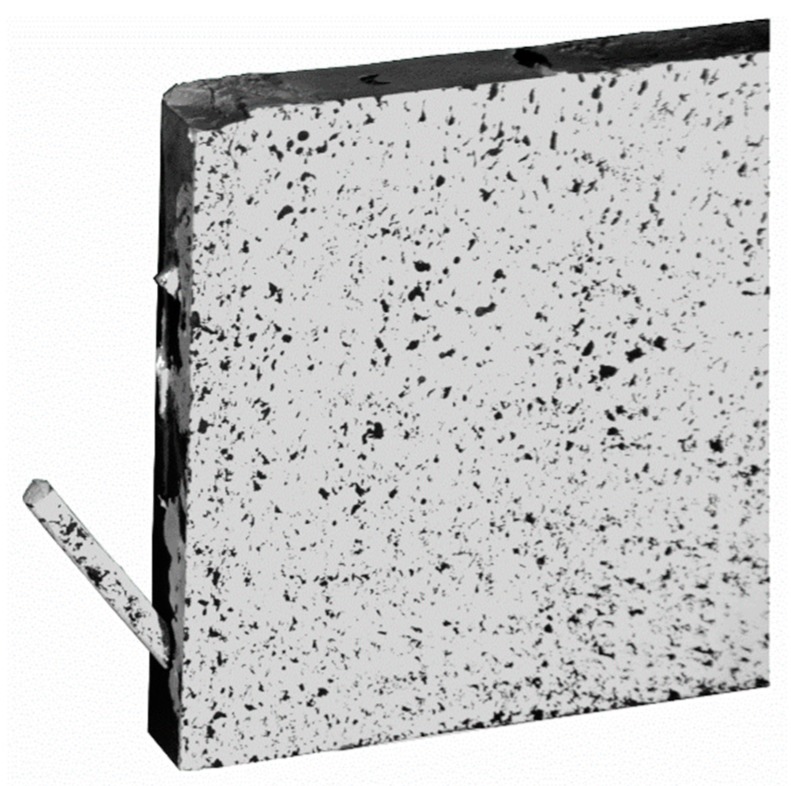
Failure mode of implant type 10—brittle failure of the compression and tension struts of the implant.

Implant Type 10 was produced in a binder-jetting process. Due to manufacturing inaccuracies, the fixation point of the implant had a smaller diameter than designed. This required a steel rod with a smaller diameter than in the other specimens to be used to mount the beam. A small gap between the implant and the steel rod led to a high concentrated loading of the mounting ring, which caused the deformation and premature brittle failure of the implant. The failed test specimen is shown in Figure 32.

## 5. Summary

This paper deals with the experimental investigations of implants for the support region of CFRP-reinforced UHPC beams, which were produced with a high degree of automation.

Starting from a previous generation of implants, the weight of the implants was reduced by FE-supported optimisation. A significant weight reduction of between 55.9% and 89.1% was achieved, while the maximum load capacity only decreased between 3.2% and 38.2%. 

Through a targeted weakening of some implants, additional insight into their failure behaviour was gained. The ductile behaviour of implant type 6 was particularly impressive, as this implant allowed a further increase in load after initial failure occurred.

All the examined manufacturing methods allowed the production of the implant designs but required various degrees of post-processing. While only little rework was required for implants produced by binder jetting, investment-cast implants required a blind hole to be drilled into their sleeve to accommodate the reinforcement. In implants manufactured by water jetting, subsequent welding of the sleeves to the implant was necessary. Inaccuracies occurred during the production such as within the water jet cut process of metal sheets with high thickness as well as during binder jetting of steel parts that have to be taken in consideration within a further optimisation of the production process.

Further research will focus on a further reduction of the material of the implant and the complete building component and an increased efficiency of the production procedure. The further reduction of material will include a consistent implementation of the principles of lightweight construction, stress-compliant guidance of the reinforcement onto the support implant and an optimisation of the topology of the beam. The further optimisation of the production procedure will focus on an optimisation of the production quality, minimising inaccuracies and efficiency regarding processing time, the required processing energy and the material use.

## Figures and Tables

**Table 1:** Production methods: used materials, geometric constraints and degree of automation.

Production Method	Material	Constraints	Degree of Automation
Water-jet cutting	Stainless steel 1.4301 Aluminium AlMgSi1	Constant cross section over depth	Numerically controlled steering of the water-jet cutting process and subsequent welding of the threaded sleeve onto the cut metal
Casting in 3D-printed mould (powder-binder jetting of plastic)	Stainless steel 1.4308 investment casting	Minimum thickness 3 mm; no blind holes; rounded edges	Numerically controlled 3D printing of the mould; casting by hand; slight post-processing by hand
Binder jetting of steel components	Stainless steel alloy 420 (60%); bronze (90% Cu, 10% Sn) (40%)	Minimum thickness 1 mm; no hollow cross sections	Numerically controlled 3D printing with slight post-processing by hand

**Table 2:** Dimensions of selected geometric features of the implants.

Implant Type (See Figure 6)	1, 4	2, 5	3, 6	7	8	9, 10
Thickness of head plate (mm)	7	3	7	7	3	7
Thickness of compression strut (mm)	5	5	3	-	-	-
Thickness of tension strut (mm)	4	4	2	-	-	-
Diameter of compression strut (mm)	-	-	-	11.5	11.5	9
Diameter of tension strut (mm)	-	-	-	11.5	11.5	9
Cross-sectional area of compression strut (mm^2^)	150	150	90	103.9	103.9	63.6
Cross-sectional area of tension strut (mm^2^)	120	120	60	103.9	103.9	63.6

**Table 3:** Production methods: used materials, geometric constraints, and degree of automation.

Base Material (Powder)	PMMA (55 µm)	PMMA (85 µm)
Binder type	Polypor B (PPB)	Polypor C (PPC)
Tensile strength	≥2.0 MPa	≥2.0 MPa
Yield strain	1%	1%
Firing temperature	700 °C	600 °C
Residual ash content	<0.01 weight %	<0.01 weight %
Especially suitable for	Investment casting, design models	Investment casting
Advantages	Sharp edges, high granularity and resolution, particle material reusable	Burns almost without residuals, particle material reusable
Disadvantages	Non-recyclable material, high production effort	Non-recyclable material, high production effort
Standard height of layer	150 µm	150 µm
Resolution	Up to 600 dpi	Up to 600 dpi
Dimensional accuracy	±0.4% (min. ±0.3 mm)	±0.4% (min. ±0.3 mm)
Curing with wax	Smooth, liquid-tight surface	Smooth, liquid-tight surface

**Table 4:** Properties of the steel–bronze components (implant Type 10), provided by the manufacturer [13].

Property	Test Method	Magnitude
Ultimate tensile strength	ASTM E8	682 MPa
0.2% offset yield strength	ASTM E8	455 MPa
Young’s modulus	ASTM E8	147,000 MPa
Yield point elongation	ASTM E8	2.3%
Density	MPIF 42	7.86 g/cm^3^
Coefficient of thermal expansion	ASTM E228	13.4 × 10^−6^ 1/K

**Table 5:** Geometry, production method, material and weight of the investigated types of implants. The information of the highest-performing implant from the first generation of implants [1] is given as reference [13].

Type	Geometry	Production Method	Material	Weight
Reference from [1], Figure 4		Laser cutting	Stainless steel 1.4301	1519 g
1–6	Rectangular struts			
1	basic	Water jetting	Stainless steel 1.4301	670 g
2	Thinner head plate			480 g
3	Reduced strut cross section			611 g
4	basic	Water jetting	Aluminium AlMgSi1	230 g
5	Thinner head plate			165 g
6	Reduced strut cross section			210 g
7–10	Circular struts			
7	basic	Investment casting	Stainless steel 1.4308	652 g
8	Thinner head plate			463 g
9	Reduced strut cross section			613 g
10	Reduced strut cross section	Binder jetting	Stainless steel alloy 420 infiltrated with bronze (90% Cu, 10% Cn)	610 g

**Table 6:** Properties of the used epoxy adhesive (Epoxy Resin: RenLam© M-1 and Hardener: Ren© HY 956).

Property	Magnitude
Mixing ratio (Resin/Hardener)	100/20
Pot life	30 min
Demoldable	24 h
Density	1.1 g/cm^3^
Viscosity at 25 °C	1200 mPas
Compressive strength	50 N/mm^2^
Flexural strength	67 N/mm^2^
Shore D hardness	84
Characteristics	Low shrinkage, high mechanical strength, high compatibility with glass fibre

**Table 7:** Dimensions of the concrete beams.

Type	Geometry at Connection with Implant (mm)			Geometry at Load Introduction Point (mm)		
	Thickness Bottom Edge	Thickness Top Edge	Height	Thickness Bottom Edge	Thickness Top Edge	Height
**Target**	**30.0**	**30.0**	**200**	**30.0**	**30.0**	**200**
1	30.3	30.2	199	29.3	28.1	200
2	30.5	30.2	200	29.2	28.8	199
3	30.2	30.4	199	30.1	30.0	201
4	30.5	30.5	200	30.7	30.8	200
5	31.0	30.5	200	31.1	30.2	200
6	30.4	30.8	199	30.1	30.7	201
7	30.0	30.0	200	27.7	30.8	200
8	30.1	30.0	200	29.6	29.0	200
9	30.3	30.6	201	31.1	32.2	201
10	30.0	30.0	200	30.1	29.3	200

**Table 8:** Ingredients of the UHPC mixture (per m^3^).

Constituent	Amount (kg)
(a) Water	153.5
(b) Superplasticiser (liquid/solid parts)	31.0 (21.7/9.3)
(c) Slump retainer (l/s)	13.8 (11/2.8)
(d) Shrinkage-reducing admixture (l/s)	6.9 (6.83/0.07)
(e) Defoamer	1.0
(f) Cement (CEM I 52.5 N C3A-free)	689.1
(g) Silica fume	172.3
(h) Inert additive	344.6
(i) Quartz sand 0.1–0.5 mm	927.5

**Table 9:** Properties of the UHPC mixture.

Property	Value
Water–cement ratio (a,b,c,d/f)	0.28
Sand, dry (dm^3^/m^3^)	350
Air content (vol%)	2
Water–binder ratio (a,b,c,d/f,g)	0.22

**Table 10:** Properties of the UHPC mixture.

Property	Value
Compressive strength (prism) (MPa)	167.5
Compressive strength (cube) (MPa)	176.3
Flexural strength (MPa)	12.1
Young’s modulus (MPa)	56,276
Ultimate compressive strain (mm/m)	4.2
Age of concrete (days)	35

**Table 11:** Properties of the carbon rods (manufacturer’s specifications).

Property	Value
Fibre material	Carbon
Impregnation material	Epoxy resin
Diameter (mm)	8
Average breaking stress (N/mm^2^)	2048
Young’s modulus (N/mm^2^)	161,000
Density (kg/m^3^)	1880

**Table 12:** Properties of the textile reinforcement (Q95/95-CCE38).

Fibre Material	Carbon
Impregnation material	Epoxy resin
Roving axis distance (mm)	38
Cross section of the strand (mm^2^)	3.62
Cross section of the reinforcement (mm^2^/m)	95
Mean breaking stress (N/mm^2^)	3112
Young’s modulus (N/mm^2^)	235,216
Elongation at break (mm/m)	13.24

**Table 13:** Maximum applied loads, maximum deflections and calculated tensile loads at the anchorage of the test specimens.

Type	1	2	3	4	5	6	7	8	9	10
Maximum load (kN)	27.4	28.0	32.1	33.8	28.6	34.2	33.2	29.3	39.7	25.5
Tensile load at anchorage (kN)	8.4	8.6	9.9	10.4	8.8	10.5	10.2	9.0	12.2	7.8
Maximum deflection (mm)	20.0	18.8	35.1	23.7	20.0	23.8	26.4	20.6	26.7	17.5

**Table 14:** Comparison of second- and first-generation implants: The maximum load and implant weight of each implant type are given as percentages of the maximum load and weight of the highest-performing first-generation implant.

Type	1	2	3	4	5	6	7	8	9	10
Maximum load	66.8%	68.3%	78.3%	82.4%	69.8%	83.4%	81.0%	71.5%	96.8%	62.2%
Implant weight	44.1%	31.6%	40.2%	15.1%	10.9%	13.8%	42.9%	30.5%	40.4%	40.2%

## References

[1] Kromoser B., Gericke O., Sobek W. (2018). Implants for load introduction into thin-walled CFRP-reinforced UHPC beams. Compos. Struct..

[2] Schlaich J., Schäfer K., Jennewein M. (1987). Toward a Consistent Design of Structural Concrete. PCI J..

[3] Sobek W., Mittelstädt J., Kobler M. (2011). Fügung schlanker Bauteile. Beton-und Stahlbetonbau.

[4] Kromoser B., Preinstorfer P., Kollegger J. (2019). Building lightweight structures with carbon-fiber-reinforced polymer-reinforced ultra-high-performance concrete: Research approach, construction materials, and conceptual design of three building components. Struct. Concr..

[5] Fédération internationale du béton (FIB) (2014). Planning and Design Handbook on Precast Building Structures, Chapter: Suitability of Precast Concrete Construction.

[6] Steinle A., Bachmann H., Tillmann M. (2016). Bauen mit Betonfertigteilen im Hochbau.

[7] Kobler M. (2013). Ein Implantat zur Einleitung Konzentrierter Lasten in Bauteile aus ultrahochfestem Beton. Ph.D. Dissertation.

[8] Mittelstädt J. (2015). Zur Einleitung Lokaler Lasten in Dünnwandige Bauteile aus Ultrahochfestem Faserfeinkornbeton Mittels Implantaten. Ph.D. Dissertation.

[9] Baumgartner A., Harzheim L., Mattheck C. (1992). SKO (soft kill option): The biological way to find an optimum structure topology. Int. J. Fatigue.

[10] Aich U., Bandyopadhyay A., Banerjee S. (2013). A State of the Art—Review on Abrasive Water Jet Machining Process. Int. Rev. Mech. Eng..

[11] Gibson D., Rosen I.W., Stucker B. (2015). Additive Manufacturing Technologies: 3D Printing, Rapid Prototyping and Direct Digital Manufacturing.

[12] Voxeljet A.G. https://www.voxeljet.com/.

[13] (2019). i. Materialise. https://i.materialise.com/.

[14] Schumann A., May M., Curbach M. (2018). Carbonstäbe im Bauwesen: Teil 1: Grundlegende Materialcharakteristiken. Beton-und Stahlbetonbau.

[15] Bergmeister K. (2005). Verstärkung mit Kohlenstofffasern—Teil 1: Verstärkung von Biegeträgern. Beton-und Stahlbetonbau.

[16] Ehlig D., Schladitz F., Frenzel M., Curbach M. (2012). Textilbeton—Ausgeführte Projekte im Überblick. Beton-und Stahlbetonbau.

[17] Preinstorfer P., Kromoser B., Kollegger J. (2019). Flexural behaviour of filigree slab elements made of carbon reinforced UHPC. Constr. Build. Mater..

[18] Sayed Ahmad F., Foret G., Le Roy R. (2011). Bond between carbon fibre-reinforced polymer (CFRP) bars and ultra high performance fibre reinforced concrete (UHPFRC): Experimental study. Constr. Build. Mater..

[19] Jesse F., Curbach M. (2010). Verstärken mit Textilbeton. Beton Kal..

